# Respiratory syncytial virus seasonality in Brazil: implications for the immunisation policy for at-risk populations

**DOI:** 10.1590/0074-02760150341

**Published:** 2016-05

**Authors:** André Ricardo Ribas Freitas, Maria Rita Donalisio

**Affiliations:** 1 Faculdade de Medicina São Leopoldo Mandic, Campinas, SP, Brasil; 2 Universidade Estadual de Campinas, Faculdade de Ciências Médicas, Campinas, SP, Brasil

**Keywords:** respiratory syncytial virus, seasonality, palivizumab, time series analysis

## Abstract

Respiratory syncytial virus (RSV) infection is the leading cause of hospitalisation for respiratory diseases among children under 5 years old. The aim of this study was to analyse RSV seasonality in the five distinct regions of Brazil using time series analysis (wavelet and Fourier series) of the following indicators: monthly positivity of the immunofluorescence reaction for RSV identified by virologic surveillance system, and rate of hospitalisations per bronchiolitis and pneumonia due to RSV in children under 5 years old (codes CID-10 J12.1, J20.5, J21.0 and J21.9). A total of 12,501 samples with 11.6% positivity for RSV (95% confidence interval 11 - 12.2), varying between 7.1 and 21.4% in the five Brazilian regions, was analysed. A strong trend for annual cycles with a stable stationary pattern in the five regions was identified through wavelet analysis of the indicators. The timing of RSV activity by Fourier analysis was similar between the two indicators analysed and showed regional differences. This study reinforces the importance of adjusting the immunisation period for high risk population with the monoclonal antibody palivizumab taking into account regional differences in seasonality of RSV.

Respiratory syncytial virus (RSV) is a non-segmented enveloped RNA virus of the family *Paramyxoviridae*, genus *Pneumovirus*. Although RSV causes upper respiratory tract diseases in patients of every age, more severe influenza-like illness occurs in children. Most children are infected during the first year of life, and almost all are infected before the age of 2 years (CDC 2014).

RSV is the leading cause of hospitalisation for respiratory diseases among children under 5 years of age, most commonly during the first year of life and almost all cases under 2 years. RSV has also been associated with asthma exacerbation, sibilance episodes, and invasive pneumococcal disease (Dulek & Peebles Jr 2011, Lotz & Peebles 2012, Régnier & Huels 2013, Weinberger et al. 2014).

The main factors associated with the severity of the infection in infants under 5 years old are prematurity, congenital heart disease, and chronic lung disease of prematurity (CID 2009). RSV reinfections throughout life are common, generally manifesting through influenza-like illness.

In Brazil, RSV was found in 23.1-42.2% of the infants hospitalised for lower respiratory tract diseases and was associated with 3.6% of the deaths (Salomão Jr et al. 2011, Lamarão et al. 2012). However, the lack of data on the role of RSV in the total number of hospitalisations and mortality by lower respiratory tract diseases in children under 5 years old, especially in developing countries, complicate the evaluation of the global impact of the disease; therefore, it is difficult to better define the priorities and investments needed for prevention and treatment (Nair et al. 2010).

In temperate climates, RSV exhibits a clear seasonal incidence pattern, occurring mostly during winter (CID 2009, Bloom-Feshbach et al. 2013). The seasonality in tropical and equatorial regions, in contrast, seems to be less marked (Piñeros et al. 2013, Stockman et al. 2013).

In Brazil, regional specificities in the seasonality of viral circulation were identified in the South (Porto Alegre) (Straliotto et al. 2002), Southeast (São Paulo) (Paiva et al. 2012), Northeast (Fortaleza) (Alonso et al. 2012) and North (Belém) (Lamarão et al. 2012) regions. However there is still no comprehensive study of seasonality across the country.

Despite improvements in RSV infection supportive care, no vaccine is available (Borchers et al. 2013, Haynes 2013). Monoclonal antibody prophylaxis has been shown to be effective in reducing the number of hospitalisations in children under 2 years of age with risk factors for aggravation (IMpact-RSV Study Group 1998). However, the high cost of prophylaxis has limited its usage, especially in developing countries. Current protocols recommend a maximum of five consecutive monthly doses of palivizumab per year during the period of greatest incidence of RSV (CID 2009, SBP 2011).

The aim of this study was to analyse the seasonal behavior of RSV and the temporal trend of hospitalisations for bronchiolitis and viral pneumonia caused by RSV in the five regions of Brazil. Identification of the periods of higher viral circulation in each region of the country will contribute to revision of the recommendations for prevention of RSV infection and the usage of immunobiologicals in the different regions of Brazil.

## MATERIALS AND METHODS

This was an ecologic study of the time series of RSV monthly positivity in samples tested using the influenza sentinel surveillance system (“SIVEP-Gripe” - SIVEP-flu), with the purpose of identifying seasonal patterns in the five regions of Brazil from 2005-2012. The temporal trend of the rates of monthly hospitalisation for bronchiolitis and pneumonia in infants under 5 years of age, in the same regions and period, was also analysed.

All five administrative regions of Brazil were chosen as local of study because they present different socioeconomic and environmental patterns which may influence on virus circulation. The climate in the Midwest region (14,993,194 inhabitants) is predominantly tropical, with a rainy season between November and March (monthly rainfall between 13.1-255.6 × 10-3 m) and temperatures ranging 22.0-26.4ºC. The Northeast region (55,794,694 inhabitants) has a semiarid climate in the interior and a tropical Atlantic climate near the coast, where the majority of the population is concentrated, with a monthly rainfall ranging 46.4-281.4 × 10-3 m and a low temperature range (monthly average of 25.1-27.3ºC). The North region (16,983,485 inhabitants) has an equatorial climate with rainfall throughout the year (monthly rainfall between 74.4-303.9 × 10-3 m) and low thermal amplitude (averages 26.3-28.0ºC). The Southeast region (84,465,579 inhabitants) has a predominantly altitude tropical climate with rainfall concentrated between November and March (monthly rainfall between 28.2-285.6 × 10-3 m) and a slightly higher thermal amplitude (monthly average between 18.7-24.6ºC). Finally, the Southern region (28,795,762 inhabitants) has a predominantly subtropical climate with rainfall distributed throughout the year (monthly rainfall between 101.1-198.9 × 10-3 m) and lower temperatures between May and September (monthly average between 14.9-23.1ºC).

*Influenza sentinel surveillance system data (SIVEP-Gripe)* - The laboratory data were obtained from SIVEP-Gripe, which has 128 sentinel surveillance units distributed throughout all the regions of Brazil. The surveillance units record at least five samples of nasophar- yngeal secretions weekly from patients with influenza-like illness (measured fever associated with sore throat and/or cough without age group distinction). Samples are processed by using indirect immunofluorescence (IIF), including tests for influenza A and B; parainfluenza 1, 2 and 3; adenovirus; and RSV. The study variables included place of residence, age, and positive and negative IIF results for RSV.

The laboratory positivity indicator was calculated using the results of the IIF reaction of the nasophar- yngeal secretion samples: monthly positivity of IIF reaction for RSV, relative to the total of monthly valid tests, i.e., excluding the results within inadequate samples (not enough biological material, improper storage, correct material in the sample) or inconclusive results (no valid results). Because in some regions the number of samples was low until 2004 (Freitas et al. 2013), samples from 2005-2012 were selected for analysis.

*Hospitalisation data* - The hospitalisation data were obtained from the Hospital Information System of the Ministry of Health, which includes all the admissions made by the Brazilian Public Health System through Hospitalisation Authorisation. These data include over 75% of all hospitalisations in Brazil. The hospitalisation causes with a possible association to RSV were selected. These are codified in the information system as RSV-associated pneumonia, RSV-associated acute bronchitis, RSV-associated acute bronchiolitis and unspecified acute bronchiolitis, with the following respective codes from the International Disease Classification, CID-10: J12.1, J20.5, J21.0 and J21.9.

The hospitalisation rates were calculated by dividing the number of admissions of infants less than 5 years old (under the aforementioned causes) by the annual population in the same age range, estimated by the Brazilian Institute of Geography and Statistics. The hospitalisation and population data were obtained from the Informatics Department of the Brazilian Public Health System.

*Time series - Wavelet and Fourier analysis* - Initially, plots were made to visualise time parameters and seasonal trends. The IIF positivity proportions of nasopharyngeal secretion from laboratory data and hospitalisation rates were analysed using the wavelet technique for identifying periodic patterns of RSV occurrence and associated morbidity. Wavelet analysis provides information on the time-scale domain for stationary and non-stationary events, allowing for simultaneous localisation of time and of high and low frequencies. Subtle time patterns of occurrence of phenomena of different nature can be detected, as well as changes in its periodicity. Wavelet analysis permits inferences regarding the way continuous variables relate to different frequencies and whether this relationship changes over time (Torrence & Compo 1998, Cazelles et al. 2007). The algorithms described by Torrence and Compo (1998), available in free software were used (Alonso & McCormick 2012). This analysis allows establishing whether a specific periodic phenomenon has a stationary frequency, a basic assumption required in Fourier analysis.

Next, the laboratory and hospitalisation data were analysed through Fourier time series decomposition using sinusoidal harmonics of the annual and semi-annual cycles. Fourier analysis considers every period to have a periodic stable frequency throughout time. This method allows analysing signals and systems through decomposition of periodic functions in convergent trigonometric series (sin and cos). In this case, it allowed identifying the time of annual and biannual peaks of the time series analysed through multiple linear regression.

The possible occurrence of seasonal heterogeneity within the administrative regions was verified through Fourier analysis of the individual hospitalisation data of each state. Since the sole purpose of this study was the analysis of seasonality, secular trends in mortality and hospitalisations were corrected by means of polynomial regression to improve the regression fit.

*Analysis of RSV transmission annual seasons* - The probable annual stations of greater RSV circulation were defined based on the laboratory-based positivity indicator of the samples. This indicator (monthly positive samples/total of monthly valid samples) was used to define annual seasons of RSV as the five consecutive months in which higher IIF positivity was observed. This definition is consistent with the prophylaxis of five consecutive monthly doses of palivizumab, as recommended by the Brazilian Pediatric Society (SBP 2011). This criterion, with minor changes, has been used before to study RSV and influenza through analysis of monthly and weekly data, and has been shown to be adequate for the study of seasonality (Chiu et al. 2002, Bloom-Feshbach et al. 2013).

Fourier and wavelets analysis were performed using Matlab (Mathworks Inc.) software EPIPOI® (Alonso & McCormick 2012). Statistical analysis was performed using SPSS v. 13.0 (SPSS Inc., Chicago, IL) software, and the plots were constructed using Microsoft Office 2013 (Microsoft Corporation). The research was approved by Research Ethics Committee of the Faculty of Medical Sciences, University of Campinas (number 909/2013).

## RESULTS

From 2005-2012, 52,261 samples of nasopharyngeal secretions were collected, of which 2,715 [5.8%; 95% confidence interval (CI) 5.6-6.0] were positive for RSV. The number of samples collected from children under 5 years old was 12,501, of which 1,373 (11.6%; 95% CI 11.0-12.2) were positive for RSV.

The ratio of positive results from IIF assays in the age group under 5 years old for each of the five regions is shown in Table I.

**TABLE I:** Positivity for respiratory syncytial virus from indirect immunofluorescence assays of samples of nasopharyngeal secretions collected by the SIVEP-GRIPE between 2005 and 2012 in infants less than 5 years old for the five administrative regions of Brazil

Region	Samples total*	Positive samples	Positivity* (%)	CI (95%)
North	4139	386	9.6	8.7 - 10.5
Northeast	4112	269	7.1	6.3 - 7.9
Midwest	876	60	7.1	5.5 - 9.0
Southeast	2848	564	21.4	19.8 - 23
South	526	94	18.8	15.6 - 22.5
Global total	12501	1373	11.61	11 - 12.2

*Number of positive samples divided by the total number of samples (positive and negative), excluding inadequate samples and samples with inconclusive results.

**Fig. 1:**
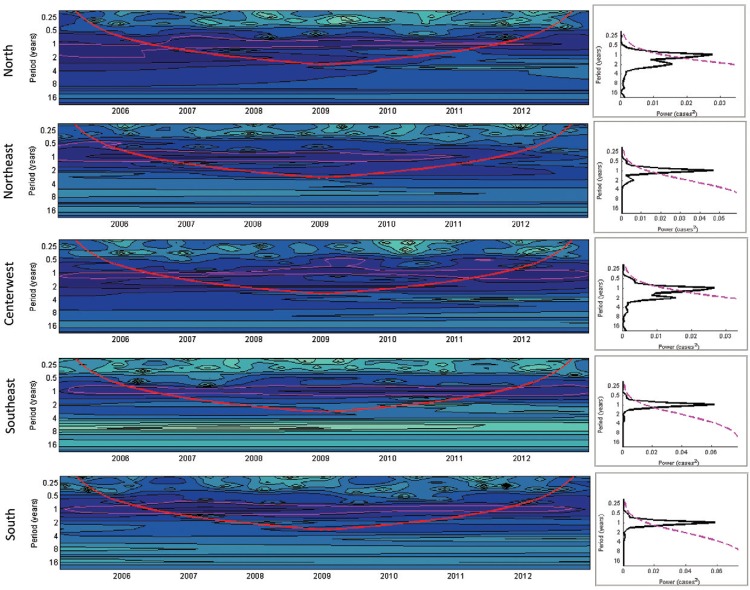
: time series of the indicator: monthly positivity of samples for respiratory syncytial virus identified by indirect immunofluorescence and divided by the number of monthly valid samples, wavelets (left), and significance spectrum (right) in the five regions of Brazil between 2005-2012. (A) Wavelet power spectra (left): darker areas correspond to higher intensity of the seasonal signal; pink contours show statistically significant areas (alpha = 5%); the red line delimits the region not influenced by the edge effects; the timescale on the left represents the time in years in an algorithmic scale of base 2. (B) Global wavelet spectrum (black line) with a significance limit of alpha = 5% (pink).

**Fig. 2:**
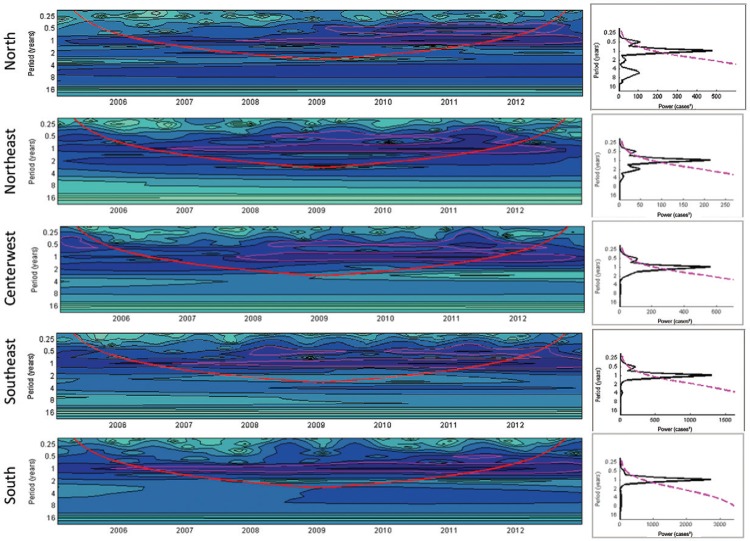
: time series of the monthly hospitalisation rates in infants under the age of 5 years due to bronchiolitis and pneumonia associated with respiratory syncytial virus (per 100,000 inhabitants), in the wavelet time series (left) and significance spectrum (right) in five regions of Brazil during 2005-2012. (A) Wavelet power spectra (left): darker areas correspond to a higher intensity of the seasonal signal; pink contours show statistically significant areas (alpha = 5%); the red line delimits the region not influenced by the edge effects; the timescale on the left represents the time in years in an algorithmic scale of base 2. (B) Global wavelet spectrum (black line) with a significance limit of alpha = 5% (pink).

**Fig. 3:**
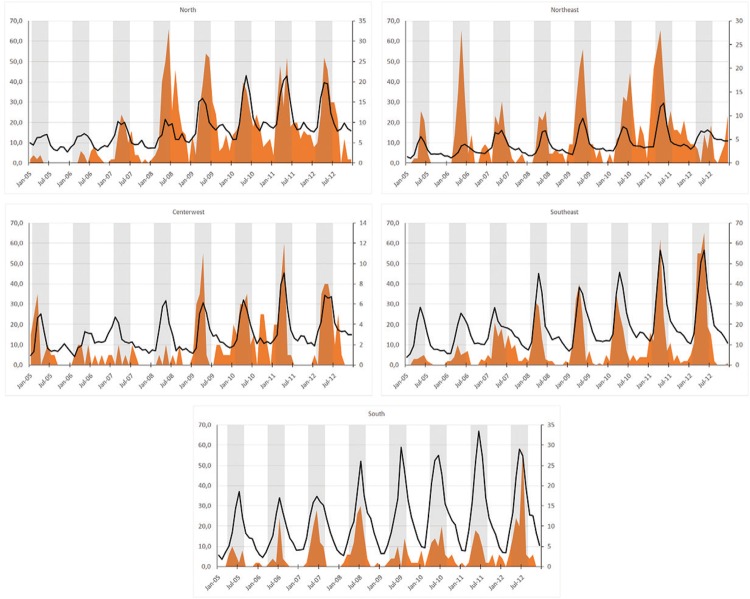
: temporal trend of sample positivity (indirect immunofluorescence) for respiratory syncytial virus, hospitalisation rates for RSV-associated bronchiolitis and pneumonia, and suggested periods for RSV infection prophylaxis with immunobiologicals for the five regions of Brazil. Jointly displays the temporal distribution of the different RSV indicators in the five regions of Brazil. The grey strips indicate the most appropriate periods for immunobiological prophylaxis in each region of the country.

*Seasonality: time series - wavelet and Fourier analysis* - Wavelet analysis permitted identifying a strong tendency for RSV annual cycles in all regions of the country; in addition one can suspect a lower second wave peak, especially in the early years of the series. It is unstable and not statistical significant, but present in Figs 1-3. This evidence may indicate a biannual (semiannual) periodicity, that is, six months after the main peak. This second peak is more evident in the North and Midwest regions in specific years (Fig. 1).

As shown in Fig. 1, there is a seasonal pattern evidenced by the wavelets. Any irregularity may be due to the small number of monthly samples.

The same annual cycle pattern with a small peak appearing after 6 months can also be observed in Fig. 2, corresponding to the hospitalisation rates for bronchiolitis and pneumonia. These stable annual activity patterns identified using wavelets allow the analysis by means of Fourier decomposition of the complete time series for years 2005-2012.

RSV seasonality analysis through Fourier decomposition of the time series assay results allowed identifying regional differences between the greater activity periods in the distinct regions of Brazil. The RSV peak occurred in the North and Midwest regions in the second week of April (peak = 4.4), in the Northeast in the first week of May (peak = 5.1), in the Southeast in the beginning of April, and in the South in the first week of June (peak = 6.2; Table II).

**TABLE II:** Month and monthly ratio of respiratory syncytial virus activity peaks following different criteria: Fourier analysis of the sample positivity, hospitalisation for bronchiolitis or pneumonia associated with RSV, and the month with the largest number of positive samples (median) for each region of Brazil during 2005-2012

Criterion (indicator)	North peak (min - max)	Northeast peak (min - max)	Midwest peak (min - max)	Southeast peak (min - max)	South peak (min - max)
Positivity monthly*	4.4	5.1	4.4	4.0	6.2
Hospitalisation^**^	4.5 (4.2 - 4.9)	5.0 (3.8 - 5.7)	4.3 (3.9 - 4.8)	4.5 (4.2 - 4.8)	6.5 (5.3 - 6.9)
Month with the highest positivity (median)^***^	4.5 (3 - 5)	5.5 (4 - 7)	4.0 (3 - 5)	4.0 (3 - 6)	7.0 (5 - 8)

In bracket - larger and smaller values found in each region. *number of monthly indirect immunofluorescence positive samples/monthly valid samples, Fourier decomposition; **rate of hospitalisation for bronchiolitis and pneumonia associated with RSV, Fourier decomposition; ***median of the indicator IIF positive samples/annual positive samples.

The seasonality Fourier decomposition analysis of the monthly rates of hospitalisation for RSV-associated bronchiolitis and pneumonia showed similar results when compared with the results from the positivity series of assays in the same region (Table II). In general, the criteria are mutually consistent, with matching results.

*Transmission peaks and annual season analysis* - The most appropriate dates for starting immunoprophylaxis with palivizumab for each region of Brazil are compared with the recent indication by Brazilian Ministry of Health (Table III). There are differences in the months suggested for the beginning of therapy with palivizumab in Southeast and Midwest region when compared with Brazilian official guidelines.

**TABLE III:** Optimal months for starting prophylaxis against respiratory syncytial virus with palivizumab, according to our indicators* and prescription of Brazilian Ministry of Health, in the five regions of Brazil

Region	Results of indicators*	Brazilian Ministry of Health 2015
North	January	January
Norwest	February	February
Midwest	January	February
Southeast	January	February
South	March	March

*positivity monthly, hospitalisation and month with the highest positivity.

## DISCUSSION

The analysis of the data collected by the SIVEP-GRIPE between the years 2005-2012 shows a clear seasonality in all regions of Brazil and regional differences between the periods of higher viral activity.

The probable RSV seasonal periods identified through samples tested by using IIF and from hospitalisation data showed similar results. Although the etiologic diagnosis of bronchiolitis is not always routinely performed in Brazilian hospitals, it is believed that RSV is responsible for 60-75% of the total number of cases of this syndrome in children under 5 years old (Nair et al. 2010). Thus we considered the diagnosis of bronchiolithis and pneumonia due to RSV as a viral circulation marker.

Several studies have demonstrated a strong correlation between the hospitalisations for bronchiolitis and pneumonia caused by RSV and the viral circulation of RSV; this includes using hospitalisation data with codes compatible with symptoms caused by RSV as a way of validating the laboratory surveillance data (Light et al. 2008, Panozzo et al. 2010, Hampp et al. 2013). The use of syndormic surveillance, besides validation of laboratory surveillance data, enables identification of underreporting or a delay in reporting, through observation of trends in the occurrence of the disease (Freitas et al. 2009, van den Wijngaard et al. 2012). Accordingly, the seasonality matching found through the laboratory and syndrome surveillance indicators are compatible and may validate the results. The secondary peaks do not seems to be artifacts.

There has been a great interest in studying RSV seasonality in order to develop appropriate prescription of palivizumab administration as well as other health care actions (Vieira et al. 2001, Paiva et al. 2012, Haynes et al. 2013). In Brazil, palivizumab has been recommended based on the first studies regarding RSV seasonality, mainly performed in the South and Southeast states (ANVISA 2011). In 2013, the Brazilian Ministry of Health included palivizumab as a drug freely distributed by the Brazilian Public Health System for use in premature babies (gestational age less or equal than 32 weeks) and infants less than 2 years old with chronic lung disease or congenital heart disease with demonstrated hemodynamic repercussion. Every state and region can establish the optimal period for starting the use of immunobiologicals, accounting for the regional climate specificities and epidemiological evidences (MS 2012). The recent revision of guidelines by the Ministry of Health in 2015 proposed a readjustment of the periods of provision of immunobiologicals in the different regions of the country (MS 2015). Our results reinforce this revision for provision of palivizumab in different regions, as suggested by others studies in Brazil (Vieira et al. 2001, Paiva et al. 2012) and others regions (Panozzo et al. 2010, Blom-Feshbach et al. 2013). Results over the period for availability of palivizumab in the Midwest and Southeast (January) do not match exactly with those suggested by Ministry of Health (February). Although there are differences of one month only, these are densely populated regions. The readjustment of the immunisation schedule with palivizumab aims to ensure the protection of higher risk children, while avoiding the waste of this expensive immunobiological.

In addition to ascertaining the optimal time for prophylaxis against RSV, scheduling of other non-pharmacological interventions can interfere with the transmission of RSV. Prevention campaigns among the population and health care providers and the planning and provision of pediatric hospital beds can be strengthened during these periods of the year (Jefferson et al. 2010, CDC 2014).

Some limitations of this study can be indicated, including the fact that the data on the positivity for RSV was obtained from an information system, which is still only partially and heterogeneously covered in various regions of the country. In addition, during the period studied, SIVEP-GRIPE did not include other viruses such as metapneumovirus, bocavirus, enterovirus, and human coronavirus, which may cause similar clinical features. Nonetheless, the analysed data represent all the information available in SIVEP-GRIPE, having a great implication on the public health policies in the country. Another limitation of this study was the low mean positivity found from the tests, which depended on several factors independent of the epidemiological situation, such as the quality of sample collection and transportation, in addition to the sensitivity of the test used. Furthermore, the number of collected samples did not allow using weekly laboratory results or the separate evaluation of the states. However, the hospitalisation data for bronchiolitis and pneumonia due to RSV did not suggest regional discrepancies.

The aggregation of samples in administrative regions can lead to inaccurate generalisations, since it does not consider differences between the various states that make up these regions. However, the reduced number of collected samples in some states did not allow this separation. Moreover, partial analysis of state data performed in this study showed similar results to those for the states aggregated into regions.

The hospitalisation rates for bronchiolitis respiratory diseases associated with RSV in children under 5 years of age can be considered an indicator that indirectly reflects the risk of circulation of RSV in the community, although there is no laboratory confirmation of these clinical conditions in the routine of hospital care in the country. The syndromic surveillance for bronchiolitis may be considered a good sentinel indicator of RSV circulation in Brazil. The results of the time series analysis of laboratory positivity indicators and hospitalisation reinforce the revision and adaptation of provision calendars for high-cost immunoprophylaxis and for RSV infection prevention campaigns in the different regions of Brazil.
